# Intensity corrections for grazing-incidence X-ray diffraction of thin films using static area detectors

**DOI:** 10.1107/S1600576724010628

**Published:** 2025-02-01

**Authors:** Fabian Gasser, Josef Simbrunner, Marten Huck, Armin Moser, Hans-Georg Steinrück, Roland Resel

**Affiliations:** a Institute of Solid State Physics Graz University of Technology Petersgasse 16 8010Graz Austria; b Department of Neuroradiology, Vascular and Interventional Radiology Medical University Graz Auenbruggerplatz 9 8036Graz Austria; c Institute for a Sustainable Hydrogen Economy (INW) Forschungszentrum Jülich Marie-Curie-Straße 5 52428Jülich Germany; d Institute of Physical Chemistry RWTH Aachen University Landoltweg 2 52074Aachen Germany; e Department of Chemistry, Paderborn University, Warburger Straße 100, 33098 Paderborn, Germany; f Anton Paar GmbH Anton-Paar-Straße 20 8054Graz Austria; Montanuniversität Leoben, Austria

**Keywords:** grazing-incidence X-ray diffraction, GIXD, intensity corrections, crystal structure solution, thin films

## Abstract

Grazing-incidence X-ray diffraction (GIXD) is the technique of choice for obtaining crystallographic information from thin films. An essential step in the evaluation of GIXD data is the extraction of peak intensities, as they are directly linked to the positions of individual atoms within the crystal unit cell. In order to obtain reliable intensities independent of the experimental setup, a variety of correction factors need to be applied to measured GIXD raw data. These include the polarization of the incident beam, solid-angle variations, absorption effects, the transmission coefficient and the Lorentz correction. The aim of this work is to provide a systematic compilation of these intensity corrections required for state-of-the-art GIXD setups with static area detectors. In a first step, analytical formulae are derived on the basis of theoretical considerations. The obtained intensity corrections are then applied to measured GIXD raw data from samples with different textures, including a single crystal and thin films containing either randomly distributed or oriented crystallites. By taking advantage of the symmetries inherent in the different types of textures, integrated peak intensities are determined, and these are compared with intensities calculated from single-crystal diffraction data from the literature. Accurate intensity corrections promise an improved quality of crystal structure solution from thin films and contribute to achieving accurate phase and texture quantifications from GIXD measurements.

## Abstract 2

Intensity correction factors for grazing-incidence X-ray diffraction are derived theoretically and applied to experimental data from thin-film samples with different textures. The provided corrections promise improved quality of crystal structure solutions from thin films and allow quantitative texture and phase analysis.

## 1. Introduction

Obtaining reliable intensities is fundamental for any type of diffraction experiment, and theories related to intensity corrections are as old as X-ray diffraction itself (Debye, 1913 ▸; Laue, 1926 ▸). For the most common diffraction experiments, such as powder diffraction and single-crystal diffraction, all required intensity corrections are well explained and documented nowadays, and sophisticated software tools including all relevant corrections are well established (Doebelin & Kleeberg, 2015 ▸; Sheldrick, 2015 ▸). In contrast, grazing-incidence X-ray diffraction (GIXD) is a relatively new technique (Marra *et al.*, 1979 ▸) with only limited analysis software available so far (Jiang, 2015 ▸; Ashiotis *et al.*, 2015 ▸; Schrode *et al.*, 2019 ▸; Reus *et al.*, 2024 ▸; Vlieg, 2000 ▸). Due to its broad range of applicability, including organic and inorganic materials of both crystalline (Sakata & Nakamura, 2013 ▸) and amorphous (Kim *et al.*, 2015 ▸; Proffit *et al.*, 2015 ▸) nature, and the growing interest in studying structures at or near surfaces, GIXD has attracted increasing attention over the past decade (Werzer *et al.*, 2024 ▸; Steele *et al.*, 2023 ▸; Wandelt, 2014 ▸).

The first step in the evaluation of GIXD data typically involves the extraction of Bragg peak positions and subsequent indexing. The indexing procedure allows determination of the lattice constants of a crystal structure (Simbrunner *et al.*, 2023 ▸). In contrast, the positions of individual atoms or the arrangement of molecules within the unit cell is linked to the intensities of the Bragg peaks via the structure factor (Als-Nielsen & McMorrow, 2011 ▸). Consequently, the determination of a complete crystal structure solution from a thin film is only achievable after obtaining reliable peak intensities from measured GIXD raw data. This is particularly important for structures that cannot be obtained as bulk crystals, because techniques like single-crystal diffraction or powder diffraction are inapplicable. Famous examples are substrate-induced or thin-film polymorphs (Jones *et al.*, 2016 ▸; Yoshida *et al.*, 2007 ▸; Gbabode *et al.*, 2012 ▸). Secondly, accurate peak intensities are essential to quantify different textures or phases within thin films (Baker *et al.*, 2010 ▸; Rivnay *et al.*, 2012 ▸; Reus *et al.*, 2022 ▸; Fischer *et al.*, 2023 ▸).

In the past, GIXD experiments were typically performed using point or line detectors in different scattering geometries (Bunk & Nielsen, 2004 ▸; Schlepütz *et al.*, 2011 ▸). Common examples are *z*-axis instruments with coupled detector and sample circles (Bloch, 1985 ▸) or vertical-axis diffractometers with independent detector and sample circles (Evans-Lutterodt & Tang, 1995 ▸; Renaud *et al.*, 1995 ▸). As it is highly important to obtain reliable intensities independent of the scattering geometry used, a considerable amount of work has been invested into determining intensity correction factors for the different experimental setups (Specht & Walker, 1993 ▸; Vlieg, 1997 ▸). Finally, geometry-independent correction factors were obtained by transforming the angular rotations of the diffractometer axes from the different scattering geometries to the same sample surface reference frame (Smilgies, 2002 ▸).

Modern GIXD setups typically use static area detectors, requiring adapted intensity corrections. Area detectors have several advantages over point or line detectors, providing access to large areas of reciprocal space in a single shot (Schlepütz *et al.*, 2005 ▸). This significantly reduces measurement times, and reliable GIXD data can be obtained for samples with limited diffracted signal while mitigating beam damage (Holton, 2009 ▸). While for a long time GIXD experiments were mainly performed at synchrotron X-ray sources, the utilization of area detectors enables large GIXD patterns to be obtained also from laboratory-based X-ray sources within reasonable timeframes (Kobayashi & Inaba, 2016 ▸; Inaba, 2017 ▸; Fischer *et al.*, 2023 ▸).

Despite the wide application of GIXD over many different fields of research, only limited information is available on intensity corrections for GIXD data from area detectors, especially for the case of thin films (Schlepütz *et al.*, 2005 ▸; Drnec *et al.*, 2014 ▸; Jiang, 2015 ▸; Reus *et al.*, 2024 ▸). In contrast, the literature mostly focuses on ultrathin layers, where crystal truncation rods (Als-Nielsen & McMorrow, 2011 ▸; Disa *et al.*, 2020 ▸) are observed in diffraction patterns. The present work aims to fill this gap by systematically compiling the different correction factors needed for GIXD experiments on thin films using static area detectors. A general guideline on how to obtain reliable intensities from such measurements is also given and the corresponding MATLAB computer code is provided at https://doi.org/10.3217/26cz1-mgs10.

## 2. Theory

The structure factor *F*_*hkl*_ describes the amplitude and phase of an X-ray beam diffracted by a crystal plane with Miller indices (*h*, *k*, *l*). Mathematically, it is calculated by
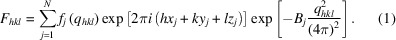
Here, *f*_*j*_ is the atomic form factor of atom *j* with fractional coordinates (*x*_*j*_, *y*_*j*_, *z*_*j*_) and atomic displacement parameter *B*_*j*_ (Alexander, 1979 ▸). *q*_*hkl*_ gives the length of the scattering vector for the Bragg peak with index *h*, *k*, *l* and is related to the scattering angle 2θ through *q* = (4π/λ)sin(2θ/2), where λ is the wavelength of the incident radiation. The last term in the equation is the Debye–Waller factor describing the reduction in diffracted radiation caused by the thermal motion of atoms. Since the thermal motion is generally anisotropic, an accurate Debye–Waller correction requires the use of an atomic displacement parameter matrix. For the purpose of this work, however, reducing the anisotropic displacement parameter matrix to an isotropic average *B*_*j*_ is sufficient (Bergmann & Taut, 2005 ▸).

A quantitative determination of measured peak intensities on an absolute scale is rather complex and requires exact knowledge of different experimental parameters which are difficult to obtain in general. Therefore, it is common practice for GIXD experiments, as well as powder diffraction and single-crystal diffraction, to use relative intensities instead. Correction factors connected to the incident angle (Robinson & Tweet, 1992 ▸) become negligible in this case, because the whole diffraction pattern would be affected by such a correction the same way. Similarly, the area factor (Smilgies, 2002 ▸; Moser, 2012 ▸; Pichler *et al.*, 2014 ▸) is not required when using two-dimensional area detectors. A rod interception factor (Feidenhans’l, 1989 ▸; Vlieg, 1997 ▸; Smilgies, 2002 ▸) is not considered for the present work because it is only required when investigating crystal truncation rods. Following these considerations, the measured intensities *I*(**q**) on a GIXD pattern are proportional to the structure factor |*F*_*hkl*_|^2^ via
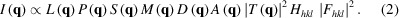
The correction factors are the **q**-dependent Lorentz correction *L*(**q**), polarization correction *P*(**q**), solid-angle correction *S*(**q**), sample–pixel distance correction *M*(**q**), detector-efficiency correction *D*(**q**), absorption correction *A*(**q**) and transmission coefficient |*T*(**q**)|^2^, and the **q**-independent peak multiplicity *H*_*hkl*_.

Applying these corrections to measured GIXD raw data *I*(**q**) allows us to compare directly the measured intensities *I*_meas_ and calculated intensities *I*_calc_ following
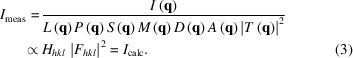
Depending on the texture of the investigated sample, the peak multiplicity *H*_*hkl*_ is required to take into account Bragg peaks with identical *q*_*hkl*_ (Als-Nielsen & McMorrow, 2011 ▸). This is important for isotropically distributed crystallites. Examples are 3D powders featuring randomly oriented crystallites, or 2D powders containing crystallites that are ordered parallel to the sample surface plane but are randomly oriented in plane (Werzer *et al.*, 2024 ▸; Fischer *et al.*, 2023 ▸). In contrast, peak multiplicities are not relevant when measuring single crystals. The specific texture of a thin-film sample can be identified via pole-figure measurements (Alexander, 1979 ▸; Heffelfinger & Burton, 1960 ▸), or by GIXD when the sample is rotated around its surface normal (Schrode *et al.*, 2019 ▸).

Most of the given correction factors such as Lorentz correction *L*(**q**), polarization correction *P*(**q**), solid-angle correction *S*(**q**), sample–pixel distance correction *M*(**q**) and detector-efficiency correction *D*(**q**) are instrument setup factors and only depend on the exact position and rotation angles of the detector with respect to the sample and the incident beam. These parameters can be obtained relatively straightforwardly by performing calibration measurements and applying software tools like *GIDVis* (Schrode *et al.*, 2019 ▸) or *pyFAI* (Ashiotis *et al.*, 2015 ▸). The correction factors depending on the sample itself are the absorption correction *A*(**q**) and the transmission coefficient |*T*(**q**)|^2^. Their applicability is consequently limited to measurements of samples with known crystal structure. However, both the absorption correction and transmission coefficient mostly affect data at small *q*_*z*_ values. In addition to the corrections shown in equation (3), it can be beneficial to apply a flat-field correction to the measured data to remove detector artefacts (Jiang, 2015 ▸; Schrode *et al.*, 2019 ▸).

In the following, detailed descriptions and derivations of all the correction factors in the denominator of the fraction in equation (3) are given for planar area detectors. We note that the formulae are typically derived in angular space and then converted to and displayed in **q** space. Details of this conversion can be found in the work of Pichler *et al.* (2014 ▸). The product of all the geometric intensity correction factors is visualized in the supporting information (Fig. S1).

### 2.1. Polarization correction

**Figure 1:**
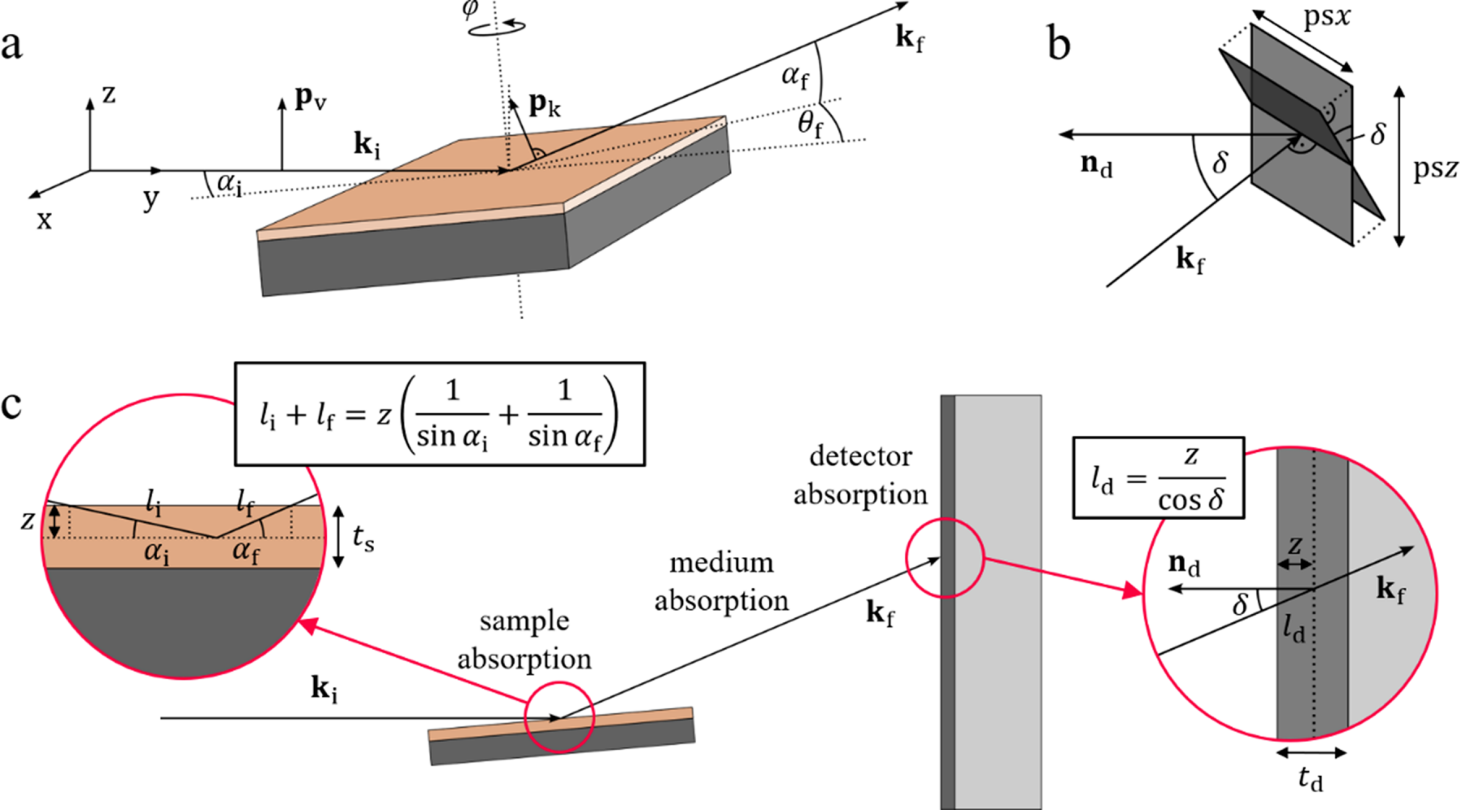
Schematics of the scattering geometry for grazing-incidence X-ray diffraction, showing the various parameters for the derivation of the intensity correction factors. (*a*) Scattering geometry including the incident wavevector **k**_i_ for an incident angle α_i_ relative to the substrate surface, the axis of sample rotation φ and the wavevector of the scattered beam **k**_f_ at the angles (α_f_, θ_f_). The normalized polarization vector **p**_v_ for a vertically polarized source and its projection **p**_k_ onto the plane perpendicular to the scattered wavevector **k**_f_ are relevant for the derivation of the polarization correction. (*b*) Detailed view of a detector pixel with dimensions ps*x* and ps*z* for the determination of the solid-angle correction. The angle between the detector normal **n**_d_ and the wavevector of the scattered beam **k**_f_ is denoted δ. (*c*) Overview of the different absorption effects related to the sample absorption (left) and to the absorption in the detector pixels (right). The thickness of the absorbing materials is *t*_s_ for the sample and *t*_d_ for the detector, and the path lengths through the sample material considering a depth *z* are given by *l*_i_ and *l*_f_ for the incident and scattered beams, respectively, and *l*_d_ on the detector.

In Thomson scattering of linearly polarized light at a single electron, the amplitude of the scattered waves is reduced depending on the scattering direction (Authier, 2013 ▸). The amount of reduction is obtained by projecting the polarization vector of the incident beam onto the plane perpendicular to the scattering direction. As depicted in Fig. 1 ▸(*a*), the normalized polarization vector of the incident X-ray beam **p**_v_ is projected towards the plane perpendicular to the scattered wavevector **k**_f_ resulting in the vector **p**_k_. Because the intensity is proportional to the square of the amplitude of the scattered wave, the intensity variations on a diffraction pattern caused by the polarization of the incident beam are given by (Smilgies, 2002 ▸)
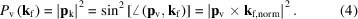
Here **k**_f,norm_ is the normalized scattered wavevector and 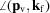 indicates the angle between the vectors **p**_v_ and **k**_f_. *P*_v_ is the polarization correction for an incident X-ray beam that is linearly polarized in the vertical *z* direction. An equivalent expression can be found for the polarization correction *P*_h_ of an incident beam that is polarized horizontally (*i.e.* in the *x* direction):
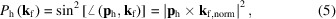
where **p**_h_ is the normalized polarization vector in the horizontal direction.

**Figure 2:**
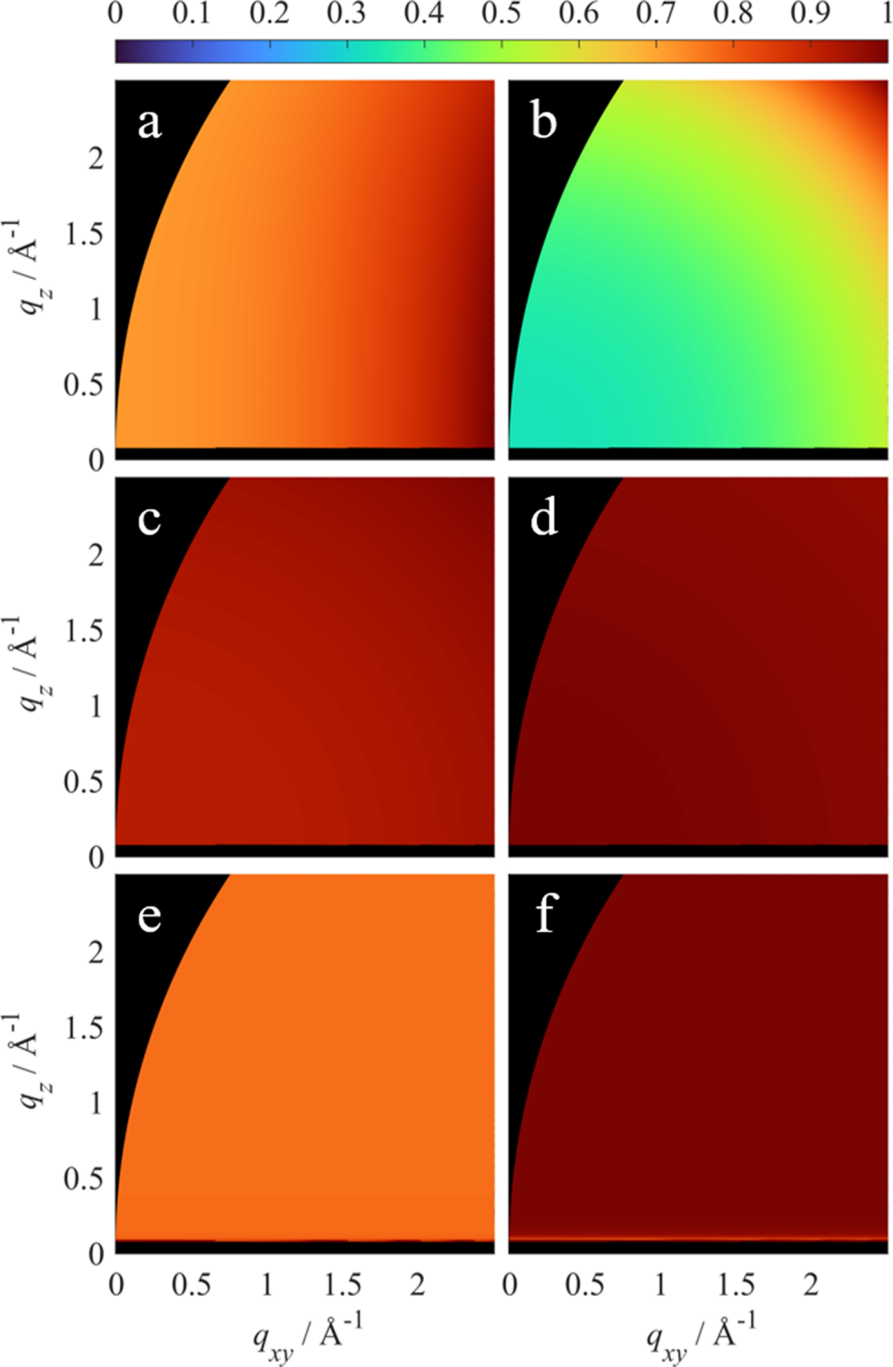
Geometric intensity correction factors in **q** space for GIXD data. Normalized inverses of (*a*) the polarization factor *P*^−1^ for ξ = 0.99, (*b*) the solid-angle correction *S*^−1^, (*c*) the sample–pixel distance correction *M*^−1^ for air as the absorbing medium, (*d*) the detector efficiency correction *D*^−1^, (*e*) the absorption correction *A*^−1^ and (*f*) the transmission coefficient |*T*|^−2^. The correction factors are calculated for an anthracene sample with a thickness of 0.34 µm and 1.4 Å X-ray radiation.

Finally, the polarization correction of an arbitrary source is described as a linear combination of the horizontal and vertical polarization corrections (Schlepütz *et al.*, 2005 ▸; Jiang, 2015 ▸):
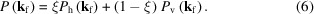
Here ξ ∈ [0, 1] is the fraction of the incident beam which is horizontally polarized. Typical values of ξ for synchrotron sources are close to 1. An example of the polarization correction with ξ = 0.99 is shown in Fig. 2 ▸(*a*). Laboratory X-ray beams generally do not have a preferential polarization direction, yielding ξ = 0.5. Inserting this value into equation (6) leads to the standard polarization correction for laboratory powder diffraction experiments *P* = 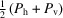 = 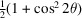, where 2θ is the scattering angle. Preferential polarization can be induced on laboratory equipment when using monochromators (Azároff, 1955 ▸; Yao & Jinno, 1982 ▸). A comparatively simple method to determine ξ of a given X-ray source is the evaluation of the intensity profile measured in transmission through a thin glass plate (Sulyanov *et al.*, 2014 ▸). Finally, particular care is required on the exact definition of ξ as various coefficients with similar but slightly different meanings are reported in the literature (Kahn *et al.*, 1982 ▸; Gilmore *et al.*, 2019 ▸).

### 2.2. Solid-angle correction

The solid-angle correction is based on the proportionality of the measured intensity on a single pixel of a detector to the solid angle covered by that pixel (Jiang, 2015 ▸). The solid angle of a given pixel is calculated by the area of the pixel projected onto the plane perpendicular to the scattered beam **k**_f_ divided by the square of the distance between the sample and the respective pixel. Consequently, the solid-angle correction *S* is given by
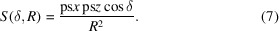
Here *R* is the sample–pixel distance, ps*x* and ps*z* are the dimensions of a detector pixel in the *x* and *z* directions, respectively, and δ is the angle between the detector normal **n**_d_ and the wavevector of the scattered beam **k**_f_ as shown in Fig. 1 ▸(*b*). The solid-angle correction is particularly important when working with large area detectors at short sample-to-detector distances. Its effect on a typical GIXD measurement is shown in Fig. 2 ▸(*b*).

### 2.3. Sample–pixel distance correction

The sample–pixel distance correction accounts for the attenuation of X-rays in the medium (typically air) between the sample and the detector. X-rays hitting the detector at larger angles α_f_ and θ_f_ pass through more of the absorbing medium and therefore undergo stronger absorption. The sample–pixel distance correction *M* simply follows from the Beer–Lambert law, giving (Jiang, 2015 ▸)
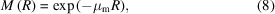
with the linear attenuation coefficient μ_m_ of the medium and the sample–pixel distance *R*. The sample–pixel distance correction is typically rather weak, as shown in Fig. 2 ▸(*c*). Its effect increases quite strongly when reducing the sample-to-detector distance. For specific setups where a vacuum is used between the sample and detector, the sample–pixel distance correction is unity.

### 2.4. Detector efficiency correction

Many of the area detectors frequently used for GIXD measurements work on a direct-detection principle. When X-rays hit the detector at oblique angles the path length through the detecting material varies, leading to an increased probability of detection (Jiang, 2015 ▸). The detector efficiency correction *D* accounts for this effect. It can be obtained by integrating the Beer–Lambert law over the path length of the scattered beam through the absorbing material of the detector as follows:
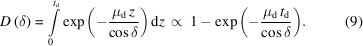
Here δ is the angle between the detector normal **n**_d_ and the scattered wavevector **k**_f_ as shown in Fig. 1 ▸(*c*, right), and *t*_d_ is the thickness and μ_d_ the linear attenuation coefficient of the sensor material of the detector. Since only relative intensities are considered, leading constants obtained by the integration are dropped for simplicity. The rather weak effect of the detector efficiency correction is shown in Fig. 2 ▸(*d*).

### 2.5. Absorption correction

Depending on the type of sample under investigation, absorption within a thin-film sample can significantly affect the obtained GIXD data (Birkholz, 2005 ▸). Due to their respective attenuation coefficients, this effect is typically stronger for inorganic thin films than for organic thin films. The absorption correction assuming a flat uniform thin film can be derived by integrating over the whole path length of the X-ray beam through the thin film [compare Fig. 1 ▸(*c*, left)]:
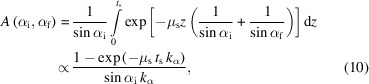
with 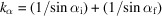. The thickness of the thin film is given by *t*_s_ and the linear attenuation coefficient of the thin-film material is μ_s_. The 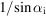 term leading the integral takes into account the beam footprint of the incident beam on the sample (Als-Nielsen & McMorrow, 2011 ▸). The absorption correction for an anthracene thin film with a thickness of 0.34 µm when using 1.4 Å X-ray radiation is shown in Fig. 2 ▸(*e*).

For samples with a large μ_s_*t*_s_ product, *i.e.* a thin-film thickness lower than the penetration depth of X-rays in the medium (Birkholz, 2005 ▸; Robinson & Tweet, 1992 ▸), equation (10) simplifies to 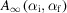 = 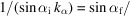

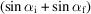
 and becomes independent of the sample properties. A significant problem with the absorption correction is the divergence of *A*^−1^ when α_f_ approaches 0. Consequently, applying an absorption correction to a measured GIXD pattern gives diverging data for small α_f_. An attempt to resolve this issue is given in the supporting information.

### 2.6. Transmission coefficient

Refraction effects caused by the interaction of X-rays with a surface lead to an increase in the measured intensity of a GIXD pattern when the incident angle α_i_ or the exit angle α_f_ are close to the critical angle of total external reflection α_c_ (Robinson & Tweet, 1992 ▸). The refraction effects involved are described using the (complex) transmissivity (Born & Wolf, 1999 ▸):
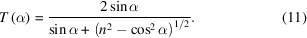
Here, *n* is the (complex) refractive index of the thin film and α is either the incident angle α_i_ or the exit angle α_f_. Since the incident angle typically remains constant throughout a GIXD measurement, an intensity correction is only required in relation to the exit angle. *T* describes the wave amplitude; the measured intensity in an experiment is consequently modified by the transmission coefficient |*T*(α_f_)|^2^ visualized in Fig. 2 ▸(*f*).

### 2.7. Lorentz correction

The Lorentz correction is a geometric correction factor required for the determination of integrated peak intensities from diffraction experiments. In its most general form, the Lorentz factor is obtained by calculating the inverse of the Jacobian *j*^−1^ for the transformation of an integral from reciprocal-space coordinates (*q*_*x*_, *q*_*y*_, *q*_*z*_) to the specific measurement coordinate frame (Specht & Walker, 1993 ▸; Evans-Lutterodt & Tang, 1995 ▸; Vlieg, 1997 ▸; Smilgies, 2002 ▸; Drnec *et al.*, 2014 ▸). Modern software tools (Schrode *et al.*, 2019 ▸; Ashiotis *et al.*, 2015 ▸) allow an easy transformation of measured GIXD data from different types of experimental setups to reciprocal-space coordinates, significantly simplifying the required Lorentz corrections. Correspondingly, the integrated intensity of a Bragg peak can be obtained in Cartesian, cylindrical or spherical reciprocal-space coordinates following
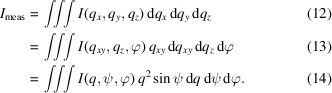
Here, (*q*_*x*_, *q*_*y*_, *q*_*z*_) are the three components of the reciprocal-space vector **q** = **k**_f_ − **k**_i_ in a Cartesian system. The radial component of cylindrical reciprocal-space coordinates is calculated using 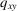 = 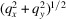 and the azimuthal angle φ via 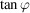 = 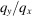. Reciprocal spherical coordinates use the same azimuthal angle φ but with radius *q* = 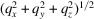 and polar angle ψ obtained from 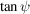 = 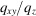.

**Figure 3:**
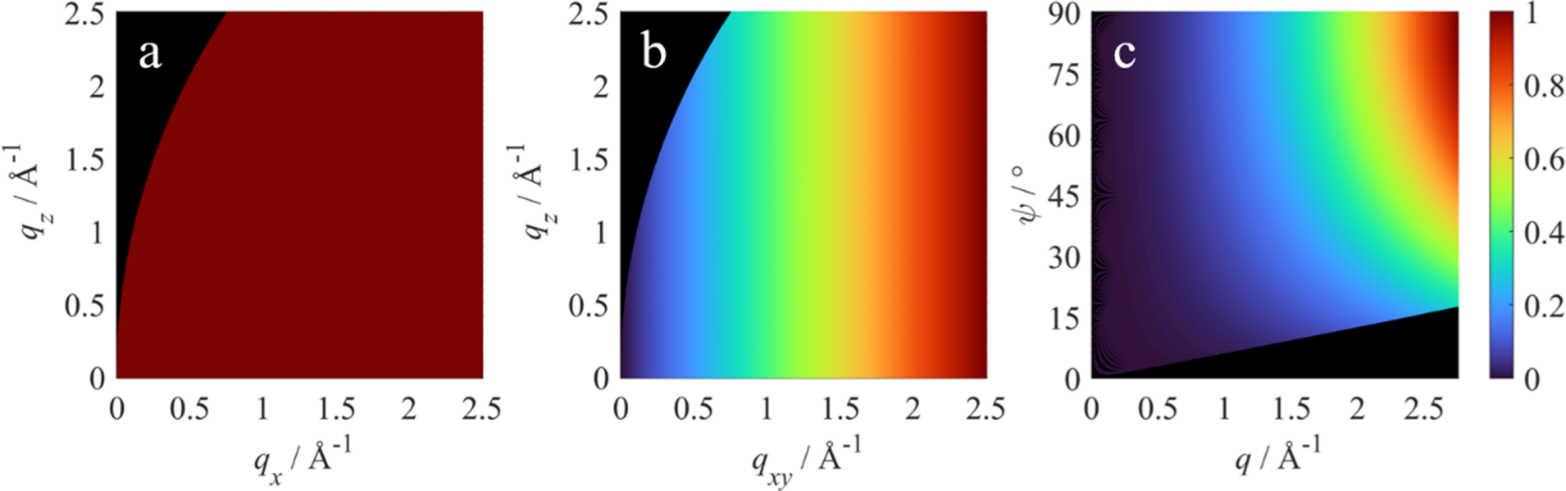
Intensity correction factors for peak integration of GIXD data. Normalized inverses of the Lorentz correction when integrating in (*a*) Cartesian reciprocal-space coordinates (*q*_*x*_, *q*_*y*_, *q*_*z*_), (*b*) cylindrical reciprocal-space coordinates (*q*_*xy*_, *q*_*z*_, φ) and (*c*) spherical reciprocal-space coordinates (*q*, ψ, φ). In every case, the Lorentz correction is independent of the unshown dimension [*i.e.q*_*y*_ for panel (*a*), and φ for panels (*b*) and (*c*)].

Depending on the data and the measured sample, it can be practical to perform peak integration in any of the given coordinate frames. The different Lorentz corrections, *i.e.L* = 1, *L* = 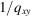 or *L* = 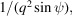 are shown in Fig. 3 ▸ in their respective coordinate frames.

## 3. Materials and methods

To verify the theoretically derived correction factors on real GIXD measurements, samples with distinct types of textures were prepared. These were 3D powders representing randomly distributed crystallites, 2D powders with a uniplanar texture and a single crystal. A detailed explanation of these textures and how they affect GIXD measurements is given by Werzer *et al.* (2024 ▸).

Lanthanum hexaboride (LaB_6_) powder was purchased from NIST (Standard 660c). The powder was mixed into a polystyrene solution and spin coated onto a silicon wafer and two glass substrates. Anthracene was bought from Tokyo Chemical Industry (TCI) with a purity of >99.5% (purified by sublimation). A thin film was prepared via drop casting from a 1 g l^−1^ tetra­hydro­furan solution onto a silicon wafer substrate. The silicon wafers used (Siegert Wafers) are atomically flat and terminated with a native oxide layer. Prior to usage, the substrates were cleaned using acetone and propan-2-ol and sonicated for 10 min in a propan-2-ol bath. Finally, the substrates were dried in a nitro­gen stream.

**Figure 4:**
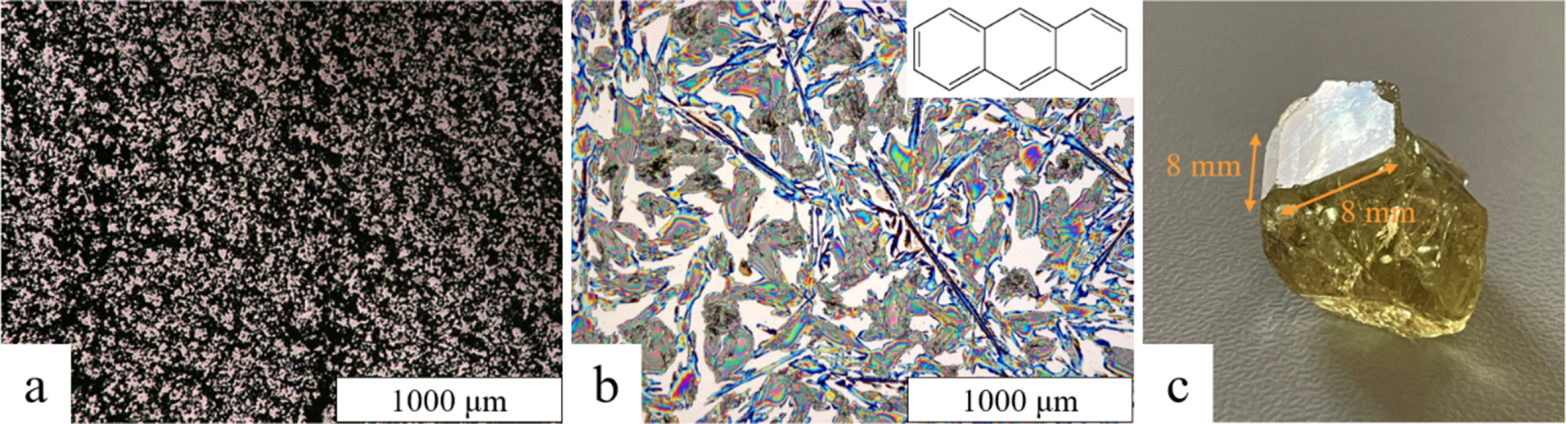
Surface structures of three of the samples under investigation. (*a*) Optical micrograph in transmission mode of an LaB_6_ thin film on a glass substrate. (*b*) Optical micrograph in reflection mode of an anthracene thin film on a silicon substrate. (*c*) Photographic image of the fluorapatite single crystal, indicating the size of the flat surface used for GIXD measurements.

Optical microphotographs of the LaB_6_ and anthracene films taken on an Olympus BX51 microscope are shown in Figs. 4 ▸(*a*) and 4 ▸(*b*), respectively. Both indicate the formation of polycrystalline inhomogeneous thin films. Thickness measurements of the polycrystalline films were performed using a KLA Tencor D-500 stylus profilometer. The average thickness of the LaB_6_ films is (6.0 ± 1.1) µm and that of the anthracene thin film is (0.34 ± 0.12) µm. The large standard deviations (18% for LaB_6_ and 35% for anthracene) confirm the in­homo­geneous nature of the thin films.

A fluorapatite single crystal was provided by Universal­museum Joanneum, Graz (inventory number 86.359). The crystal has a flat surface with a size of 8 × 8 mm; a photograph is shown in Fig. 4 ▸(*c*).

GIXD measurements were performed on the XRD1 beamline, Elettra Sincrotrone (Trieste, Italy). The synchrotron radiation used, with a wavelength of 1.4 Å, is polarized horizontally (*i.e.* in the plane of the electron storage ring) with ξ = 0.99 checked following Sulyanov *et al.* (2014 ▸) (see the supporting information). The LaB_6_ thin films and the fluorapatite single crystal were measured with a 100 µm pinhole at an incident angle of α_i_ = 1°. The anthracene thin film was measured with a 200 µm pinhole at an incident angle of α_i_ = 0.65° to achieve a larger beam footprint on the sample and therefore improved statistics. All incident angles used are significantly above the critical angles of the investigated materials (α_c_ = 0.26° for LaB_6_, α_c_ = 0.15° for anthracene and α_c_ = 0.24° for fluorapatite). For collecting the raw GIXD intensities, a Dectris Pilatus2M detector was used at a nominal sample-to-detector distance of 20 cm. All measurements were performed while rotating the sample around its surface normal for 360° indicated by the rotation angle φ [compare Fig. 1 ▸(*a*)]. For LaB_6_ and anthracene, images of individual rotation steps were summed for improved statistics. For the fluorapatite single crystal, 720 measurements were performed while rotating the sample for 0.5° for every measurement. For calibration an LaB_6_ standard was used. The measured data were transformed to reciprocal space using the software *GIDVis* (Schrode *et al.*, 2019 ▸). Further data processing and evaluation were performed in MATLAB. The corresponding MATLAB code is provided together with the GIXD raw data at https://doi.org/10.3217/26cz1-mgs10.

To compare measured and calculated intensities a reliability factor was calculated:
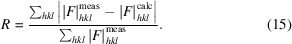
Here, 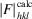 gives the calculated structure factor and 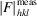 indicates the measured structure factor given by the square root of the measured intensity *I*_meas_ after application of all the correction factors.

## 4. Results

### 4.1. Randomly distributed crystallites

**Figure 5:**
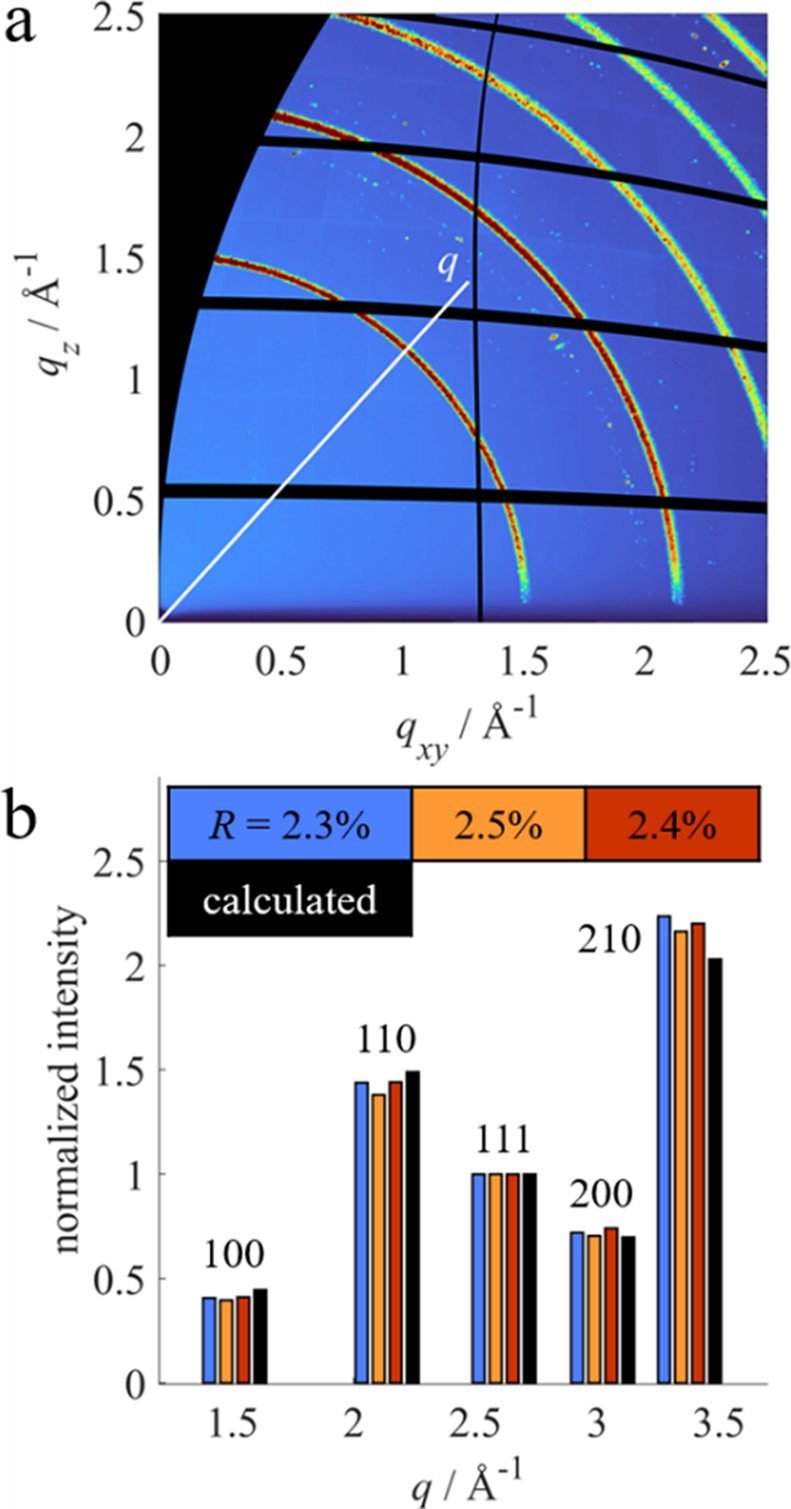
GIXD measurement of an LaB_6_ thin film. (*a*) Measured GIXD pattern before application of the correction factors, showing Debye–Scherrer rings. (*b*) Histogram comparing measured peak intensities *I*_meas_ (blue: silicon substrate; orange and red: glass substrate) after application of the correction factors and calculated intensities *I*_calc_ (black) from single-crystal data. The histogram bars are centred around the *q* position of their respective Bragg peak and normalized with respect to the 111 peak. Reliability factors *R* for each measurement are given in the legend.

The measured (uncorrected) GIXD pattern of an LaB_6_ thin film on a silicon substrate is shown in Fig. 5 ▸(*a*). Instead of distinct diffractions spots, Debye–Scherrer rings are visible, indicating randomly distributed crystallites (3D powder). The black areas in the GIXD pattern originate from the experimentally inaccessible missing wedge (Werzer *et al.*, 2024 ▸) and detector blind spots. Measured intensities along the Debye–Scherrer rings are almost constant (variation below 10%), suggesting no preferred orientation of the LaB_6_ crystallites.

Towards small *q*_*z*_ values a drop in intensity is clearly visible in the GIXD pattern. This effect diminishes after application of the different intensity corrections; in particular, the absorption correction has strong effects in this regime.

Accurate peak intensities from a 3D powder can be obtained by extracting a line profile *I*(*q*) in any direction of the intensity-corrected GIXD pattern. Consequently, the line profile was taken from a regime at higher *q*_*z*_, where absorption and roughness effects are expected to play only a minor role. After fitting and removal of the background, numerical peak integration was performed. Assuming a spherical symmetry as expected for a 3D powder, equation (14) simplifies to
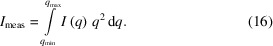


The obtained integrated intensities *I*_meas_ for the different peaks are shown in Fig. 5 ▸(*b*), represented by the heights of the blue, orange and red histogram bars for three different measurements: blue corresponds to the measurement on the silicon substrate, and orange and red show the results of additional measurements on glass substrates. All shown intensities were normalized with respect to their 111 peak. The black histogram bars represent calculated values *I*_calc_ from single-crystal diffraction data (Eliseev *et al.*, 1986 ▸) following equation (1). The calculation includes a Debye–Waller factor using isotropic averaged atomic displacement parameters, and peak multiplicities were taken into account. In this sense, Fig. 5 ▸(*b*) gives a comparison between the left- and right-hand sides of equation (3). Tables including the full list of all the measured and calculated intensities for all the samples are given in the supporting information (Tables S1–S3).

The measured peak intensities show good agreement with the calculated reference intensities, leading to reliability factors below 2.5% independent of the substrate used. Despite the roughness of the films, using an absorption correction assuming a flat homogeneous film still significantly improves the obtained reliability factor by 0.5% on average compared with data that were not absorption corrected.

### 4.2. Uniplanar textured thin film

**Figure 6:**
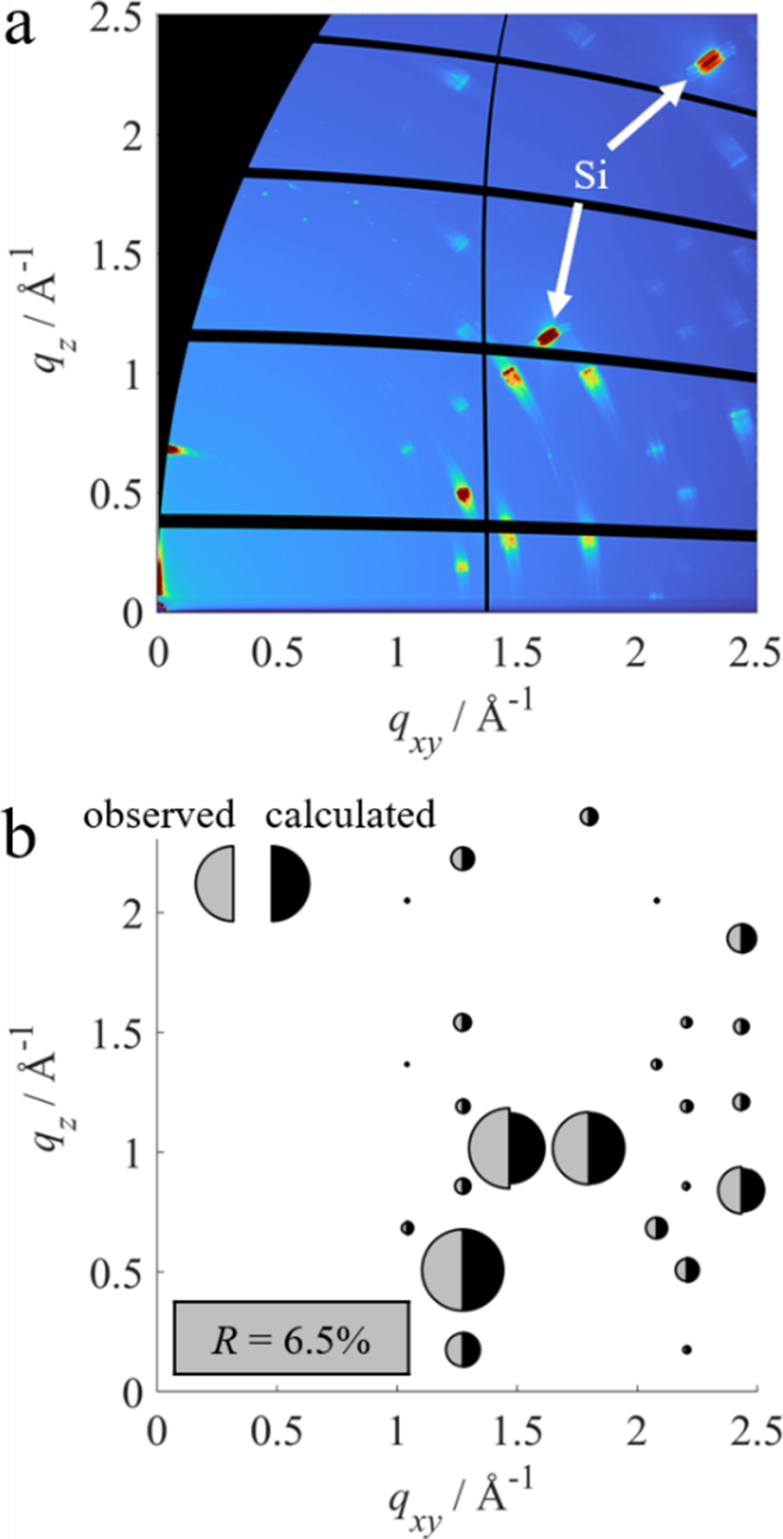
GIXD measurement of an anthracene thin film. (*a*) Measured GIXD pattern, showing distinct diffraction peaks of anthracene with (001) orientation. The peaks indicated with white arrows correspond to the silicon substrate. (*b*) Comparison between measured peak intensities *I*_meas_ (grey areas) and calculated intensities *I*_calc_ (black areas) centred around their respective positions in reciprocal space. Intensities are normalized with respect to the 110 peak. The reliability factor *R* is given in the grey box.

Fig. 6 ▸(*a*) shows the measured GIXD pattern of an anthracene thin film. Distinct diffraction spots are observed, as expected for a thin film with uniplanar (2D powder) texture. This means that the individual anthracene crystallites are oriented with the (001) plane parallel to the substrate surface but do not have any preferred azimuthal orientation. Individual slightly misoriented crystals lead to a lateral smearing of the diffraction peaks, denoted as out-of-plane mosaicity. At the lower edge of the GIXD pattern the enhanced intensity caused by the transmission coefficient is observed. Additional to the anthracene peaks, two peaks originating from the silicon substrate are clearly visible, marked by white arrows.

For peak integration, the intensity-corrected GIXD data were transformed into spherical reciprocal-space coordinates. For every anthracene peak the background was removed and numerical 2D integration was performed using
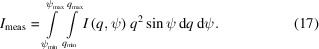
The integration limits [*q*_min_, *q*_max_] and [ψ_min_, ψ_max_] were chosen manually for each peak in order to include as much intensity as possible from the respective peak while minimizing overlaps. Peaks significantly influenced by detector blind spots were not taken into consideration. The obtained integrated intensities *I*_meas_ are shown in Fig. 6 ▸(*b*) in the form of the areas of the grey half-circles. For comparison, the areas of the black half-circles correspond to the calculated intensities *I*_calc_ obtained from single-crystal diffraction data (Mason, 1964 ▸), including peak multiplicities but without a Debye–Waller factor. The circles are centred around the position of the respective Bragg peak in the measurement and are normalized with respect to the 110 peak.

Measured and calculated intensities show excellent agreement, leading to a reliability factor of 6.5%. Only minor deviations are visible without the presence of any trends, indicating reliable intensity corrections. A comparable measurement was performed on the same thin film but using a smaller pinhole and higher incident angle. The results show a similar very good agreement, in this case with a reliability factor of 8.8%. The higher reliability factor can be attributed to the weaker statistics of the measurement caused by the smaller beam footprint on the sample. A full list of all the intensities obtained from both measurements is given in the supporting information (Tables S4 and S5).

### 4.3. Single crystal

**Figure 7:**
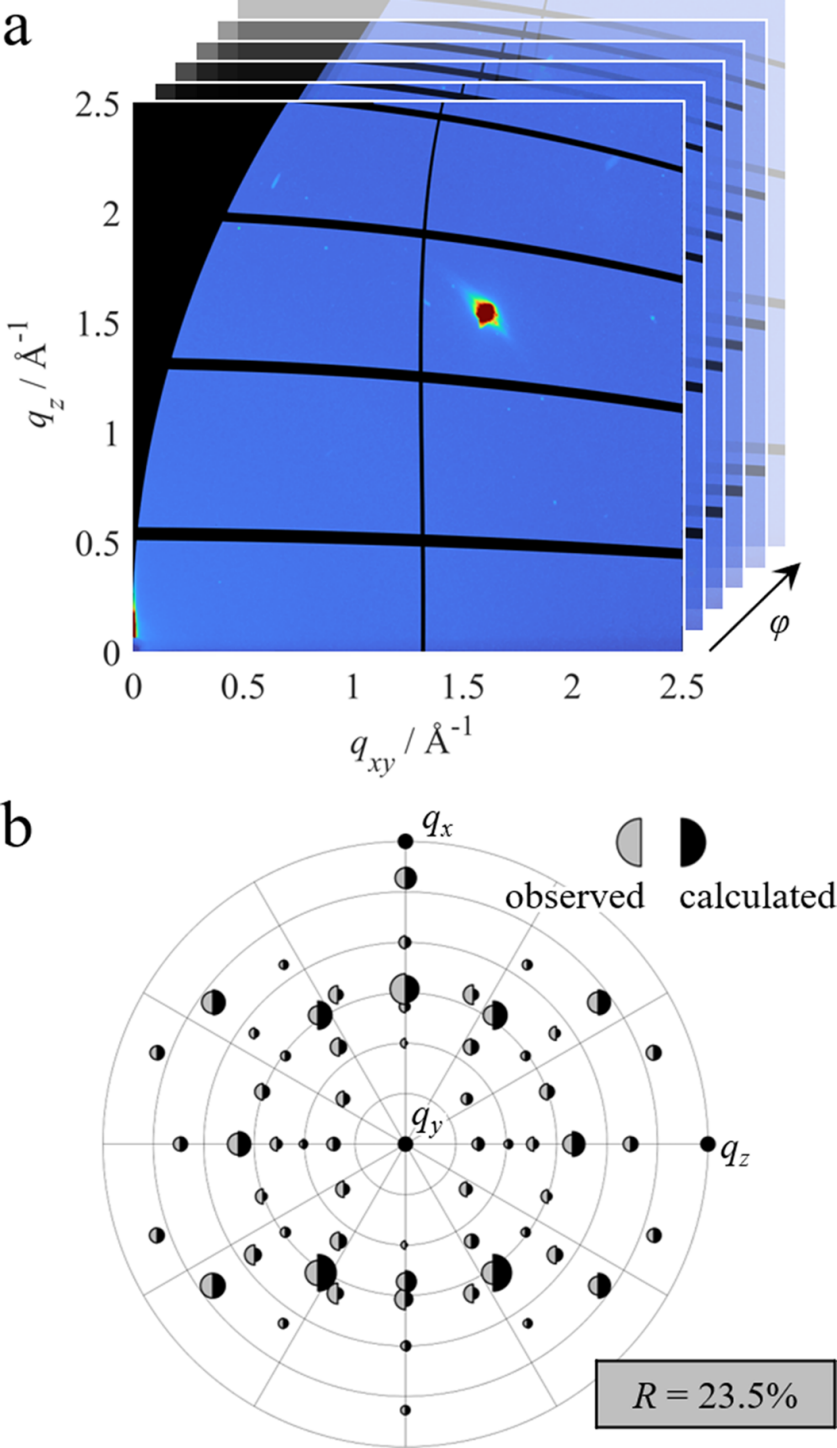
GIXD measurement of a fluorapatite single crystal. (*a*) A single GIXD pattern only shows a single Bragg peak, emphasizing the necessity of sample rotation φ. (*b*) Comparison between measured peak intensities after correction *I*_meas_ (grey areas) and calculated intensities *I*_calc_ (black areas) at their respective positions in the stereogram. Intensities are normalized with respect to the 111 peak. The reliability factor *R* is given in the grey box.

Fig. 7 ▸(*a*) shows one of the 720 measured GIXD patterns for the fluorapatite single crystal. Only a single diffraction spot is visible due to the single-crystalline nature of the sample. A detailed analysis of the position of the different Bragg peaks from all the measured GIXD patterns revealed that the surface of investigation of the crystal corresponds to the (010) crystalline plane.

For peak integration, intensity corrections were applied to each of the 720 measurements. A background subtraction was performed for every peak and every measurement individually. The full data set was then transformed to spherical reciprocal-space coordinates and numerical 3D integration was performed following
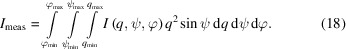
Again, all integration limits [*q*_min_, *q*_max_], [ψ_min_, ψ_max_] and [φ_min_, φ_max_] were chosen manually for each individual peak. The obtained integrated intensities *I*_meas_ are shown in Fig. 7 ▸(*b*) in the form of the areas of the grey half-circles in a stereographic projection. A full list of all peak intensities is given in the supporting information, including a few peaks which could not be visualized due to overlaps. The areas of the black half-circles in the stereogram correspond to the calculated intensities *I*_calc_ obtained from single-crystal diffraction data (Sudarsanan *et al.*, 1972 ▸) including isotropic averaged dis­place­ment parameters.

The measured and calculated intensities are in good agreement. However, the reliability factor in this case is significantly higher at 23.5%. The higher deviation could be related to difficulties with sample alignment and the influence of the beam footprint, as discussed in the following section.

## 5. Discussion

A notable problem when applying any intensity corrections to experimental data is related to the background. Together with the observed diffraction features, the background of measured GIXD data is also affected by the correction factors. In some cases, this can introduce unphysical artefacts that require particular attention. Reducing the air scattering of measured GIXD data utilizing slits or collimators can help (Kowarik *et al.*, 2019 ▸), but artefacts can only be completely avoided by fitting and removal of the background before the application of any correction factors. A complete background subtraction is difficult, as the background has a rather complex shape in general (Werzer *et al.*, 2024 ▸). Subtracting the background locally around specific diffraction features, as done in this work, might therefore be more applicable.

An effective intensity correction of measured GIXD data is only possible after precise alignment of a thin-film sample in the centre of the goniometer of the experimental setup. In particular, both the absorption correction and transmission coefficient require exact knowledge of the sample position with respect to the incident beam. Any small misalignments will make such corrections impractical (Savikhin *et al.*, 2020 ▸).

The Lorentz correction described here is applied to data that were transformed to reciprocal space. Misalignments generally lead to shifts in the position of experimentally observed Bragg peaks (Holzer *et al.*, 2022 ▸), leading to an incorrect Lorentz correction for those peaks. Deviating peak positions are also observed as a consequence of multiple scattering (Resel *et al.*, 2016 ▸; Savikhin *et al.*, 2020 ▸), making an effective Lorentz correction rather complex in such cases.

Finally, for this work it was expected that no area correction would be required, as the footprint of the incident beam on the sample is projected onto the area detector equally for every observed Bragg peak. While this is true for a static GIXD measurement, rotation of the sample around its surface normal might make an area correction necessary. If the incident beam spills over the edges of the sample surface and the sample surface is not circular, the beam footprint will change upon rotation (Schlepütz *et al.*, 2005 ▸). For this work, sample sizes and beam footprints were chosen carefully to ensure that the full beam footprint remained on the sample surface throughout the sample rotation.

## 6. Conclusion

It was the purpose of this work to provide detailed and systematic insight into the intensity corrections required for GIXD experiments on thin films using static area detectors. Analytical equations have been derived for the polarization factor, solid-angle correction, sample–pixel distance correction, detector efficiency correction, absorption correction, transmission coefficient and Lorentz correction.

The different correction factors were applied to experimental GIXD data obtained from samples with different types of textures, including 3D and 2D powder thin films and a single crystal. The measured peak intensities were compared with calculated intensities from single-crystal diffraction data and reliability factors were calculated to indicate the level of agreement. Both 3D powder and 2D powder show excellent results leading to reliability factors of 2.3% and 6.5%, respectively. The reliability factor of the single-crystal GIXD measurements is larger at 23.5%. In this sense, the work also provides an estimate of the accuracy and reproducibility of peak intensities from state-of-the-art GIXD experiments.

The presented intensity corrections contribute significantly to advanced data treatment for GIXD. Reliable peak intensities are essential for the determination of crystal structure solutions from thin films. Further applications include quantitative texture and phase analysis on thin films.

## Supplementary Material

Supporting information including some short additional explanations and tables containing detailed information about the evaluated peak intensities. DOI: 10.1107/S1600576724010628/xx5064sup1.pdf

Grazing-incidence X-ray diffraction data of samples with different textures and MATLAB code for their evaluation: https://doi.org/10.3217/26cz1-mgs10
