# Supplementary material for: Intensity corrections for grazing-incidence X-ray diffraction of thin films using static area detectors
Source: J Appl Crystallogr. 2025 Feb 1;58(Pt 1):96–106. doi: 10.1107/S1600576724010628 (PMC11798510; doi:10.1107/S1600576724010628)
Supplement: Supplementary file 1 [file j-58-00096-sup1.pdf]

JOURNAL OF
APPLIED
CRYSTALLOGRAPHY

Volume 58 (2025)

Supporting information for article:

Intensity corrections for grazing-incidence X-ray diffraction of thin films using static area detectors

Fabian Gasser, Josef Simbrunner, Marten Huck, Armin Moser, Hans-Georg Steinrück and Roland Resel

S1. Product of the geometric intensity correction factors

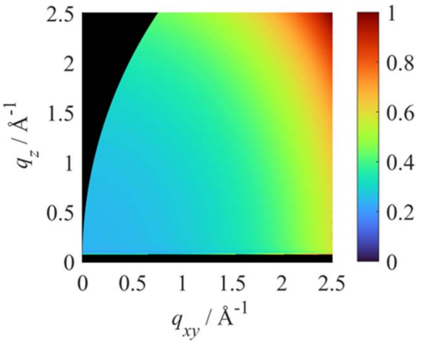

Figure S1 Normalized product of the geometric intensity correction factors in **q**-space for grazing-incidence X-ray diffraction data including the polarization factor P^{-1} for $\xi = 0.99$, the solid angle correction S^{-1}, the sample-pixel distance correction M^{-1} for air as absorbing medium, the detector efficiency correction D^{-1}, the absorption correction A^{-1} and the transmission coefficient $|T|^{-2}$. The last two correction factors are calculated for an anthracene sample with a thickness of 0.34 µm.

S2. Absorption correction for small exit angles α_f

The absorption correction A given in Eq. 10 drops to 0 when α_f approaches 0. As it accounts for infinite absorption, applying the absorption correction A^{-1} to measured GIXD data gives diverging data at small α_f. An attempt to resolve this issue can be made by limiting the size of the absorbing medium. Assuming a thin film with lateral length l_s and the incident beam hitting the centre of the sample, the absorption correction A_0 for the limiting case of small α_f can be derived following:

$$A_0 = \frac{1}{\sin\alpha_\mathrm{i}} e^{-\frac{1}{2}\mu_\mathrm{s} l_\mathrm{s}} \int_0^{t_\mathrm{s}} e^{-\mu_\mathrm{s}\frac{z}{\sin\alpha_\mathrm{i}}}\, dz \propto e^{-\frac{1}{2}\mu_\mathrm{s} l_\mathrm{s}} \left(1 - e^{-\mu_\mathrm{s}\frac{t_\mathrm{s}}{\sin\alpha_\mathrm{i}}}\right) \qquad (19)$$

This expression can be used to limit the absorption correction to not drop to 0 and consequently avoid divergence of the corrected GIXD data.

S3. Measurement of the polarization of an X-ray source

In order to perform an accurate polarization correction, knowledge on the coefficient ξ of a given X-ray source is required. A comparatively simple method to obtain this parameter involves measuring the scattered intensity through a thin glass plate (Sulyanov *et al.*, 2014). Fig. S2(a) shows the obtained diffraction signal when shooting an X-ray beam through two attached glass cover slides with a thickness of 0.15 mm each. The measurement features a broad ring caused by the amorphous nature of the glass slides. Along this ring, a slight intensity variation is visible, caused by the polarization of the incident beam. The maximum intensity along the ring as a function of the polar angle ψ is shown in Fig. S2(b). A fit was performed through the measured data points by applying (Kahn *et al.*, 1982; Sulyanov *et al.*, 2014):

$$I(\psi, 2\theta) = I_0 \left[1 - \zeta\ \cos(2\psi) \frac{\sin^2 2\theta}{1+\cos^2 2\theta}\right] \qquad (20)$$

Here, I_0 is the intensity of the incident X-ray beam, ψ is the polar angle, 2θ is the scattering angle and ζ is the polarization coefficient. The polarization coefficient ζ used here follows a different definition compared to the parameter ξ from Eq. 6. However, the two coefficients can easily be transformed into each other following:

$$\xi = \frac{\zeta+1}{2} \qquad (21)$$

For the measurement shown in Fig. S2 a polarization coefficient $\xi > 0.97$ was obtained, confirming the expected value for a synchrotron X-ray source. An comparable measurement was performed using 3 equivalent glass cover slides leading to $\xi > 0.98$ for the same source indicating good reproducibility.

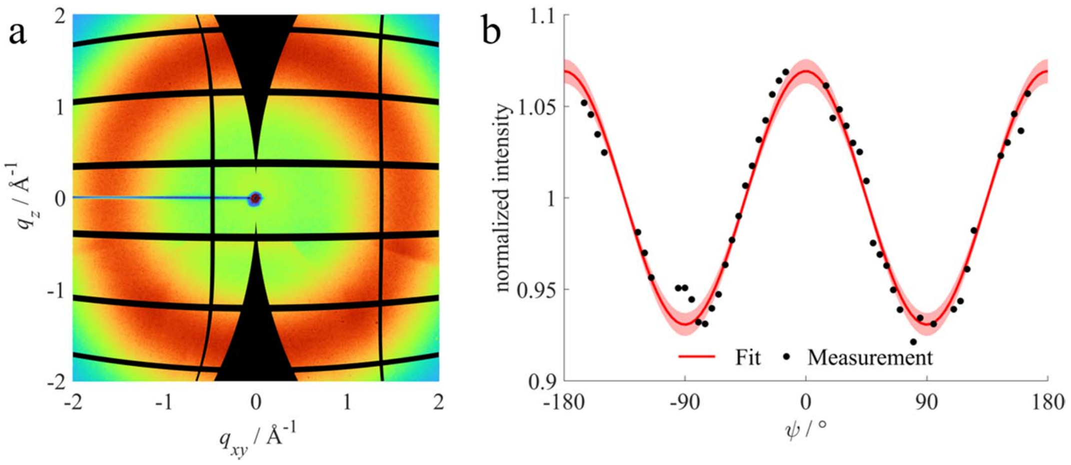

Figure S2 X-ray diffraction measurement in transmission through two attached glass cover slides: (a) Diffraction pattern showing a broad diffraction ring. The shadow of the beam stop is visible on the left side of the measurement. (b) Maximum intensity along the ring as a function of the polar angle ψ normalized with respect to the intensity of the incident X-ray beam I_0.

S4. Results LaB_6

Table S1 Table containing a full list of the calculated intensities I_{calc} of LaB_6 including the Laue indices *hkl* with peak multiplicity H_{hkl} and the reference position in q.

The measured intensities I_{meas} were obtained via 1D numerical integration with integration error ΔI_{meas} from a GIXD measurement of an LaB_6 thin film on a silicon substrate with incidence angle 1° and a 100 µm pinhole. The results lead to a reliability factor of R = 2.3%.

hkl	q / Å	H_{hkl}	I_{calc} / a.u.	I_{meas} / a.u.	ΔI_{meas} / a.u.
100	1.511	6	1423.05	72587	266
110	2.138	12	2376.22	256264	315
111	2.618	8	2392.24	178075	315
200	3.023	6	2227.77	128246	317
210	3.380	24	1618.58	398148	331

Table S2 Table containing a full list of the calculated intensities I_{calc} of LaB_6 including the Laue indices *hkl* with peak multiplicity H_{hkl} and the reference position in q.

The measured intensities I_{meas} were obtained via 1D numerical integration with integration error ΔI_{meas} from a GIXD measurement of an LaB_6 thin film on a glass substrate with incidence angle 1° and a 100 µm pinhole. The results lead to a reliability factor of R = 2.5%.

hkl	q / Å	H_{hkl}	I_{calc} / a.u.	I_{meas} / a.u.	ΔI_{meas} / a.u.
100	1.511	6	1423.05	43130	160
110	2.138	12	2376.22	150122	190
111	2.618	8	2392.24	108774	190
200	3.023	6	2227.77	76659	191
210	3.380	24	1618.58	235291	200

Table S3 Table containing a full list of the calculated intensities I_{calc} of LaB_6 including the Laue indices *hkl* with peak multiplicity H_{hkl} and the reference position in q. The measured intensities I_{meas} were obtained via 1D numerical integration with integration error ΔI_{meas} from a GIXD measurement of an LaB_6 thin film on another glass substrate with incidence angle 1° and a 100 µm pinhole. The results lead to a reliability factor of R = 2.4%.

hkl	q / Å	H_{hkl}	I_{calc} / a.u.	I_{meas} / a.u.	ΔI_{meas} / a.u.
100	1.511	6	1423.05	30924	99
110	2.138	12	2376.22	108299	117
111	2.618	8	2392.24	75136	117
200	3.023	6	2227.77	55694	118
210	3.380	24	1618.58	165365	123

S5. Results anthracene

Table S4 Table containing a full list of the calculated intensities I_{calc} of anthracene including the Laue indices *hkl* with peak multiplicity H_{hkl} and the reference position in q and ψ.

The measured intensities I_{meas} were obtained via 2D numerical integration with integration error ΔI_{meas} from a GIXD measurement of an anthracene thin film on a silicon substrate with incidence angle 0.65° and a 200 µm pinhole. The results lead to a reliability factor of R = 6.5%.

hkl	q / Å	ψ / °	H_{hkl}	I_{calc} / a.u.	I_{meas} / a.u.	ΔI_{meas} / a.u.
-111	1.285	82.16	2	631.75	83668	8367
-112	1.536	56.01	2	133.61	19293	1929
-121	2.214	85.46	2	30.13	4020	402
-122	2.368	68.74	2	20.51	5168	517
011	1.245	56.71	2	88.99	8814	881
012	1.718	37.29	2	7.36	827	83
021	2.191	71.82	2	243.16	36678	3668
022	2.490	56.7	2	49.27	7783	778
110	1.371	68.25	2	3534.19	532659	53266
111	1.744	46.90	2	109.04	14054	1405
120	2.265	77.033	2	314.14	40156	4016
121	2.508	61.63	2	75.90	11425	1143
200 + -203	1.785	55.30	1	5271.54	521353	52135
210 + -213	2.066	60.54	2	2689.77	421483	42148
-314	2.719	63.60	2	124.83	22024	2202
-315	3.084	52.15	2	451.24	63266	6327
-215 + 212	3.000	36.85	2	163.56	21863	2186
-224 + 211	3.071	56.01	2	640.55	94762	9476
-225 + 222	3.500	46.69	2	383.16	4543	4543
-113	2.000	39.55	2	149.35	24469	2447
-114	2.564	29.78	2	278.68	41995	4200
-123	2.692	55.06	2	54.02	9050	905
013	2.299	26.91	2	10.89	972	97
014	2.925	20.84	2	114.00	21096	2110
023	2.921	45.43	2	10.16	1125	113
113	2.858	26.46	2	32.79	2955	295
31-1	2.576	70.95	2	974.51	174409	17441
310	2.873	57.95	2	109.60	18257	1826

Table S5 Table containing a full list of the calculated intensities I_{calc} of anthracene including the Laue indices *hkl* with peak multiplicity H_{hkl} and the reference position in q and ψ.

The measured intensities I_{meas} were obtained via 2D numerical integration with integration error ΔI_{meas} from a GIXD measurement of an anthracene thin film on a silicon substrate with incidence angle 1° and a 100 µm pinhole. The results lead to a reliability factor of R = 8.8%.

hkl	q / Å	ψ / °	H_{hkl}	I_{calc} / a.u.	I_{meas} / a.u.	ΔI_{meas} / a.u.
-111	1.285	82.17	2	631.75	4832	483
-112	1.536	56.01	2	133.61	1813	181
-121	2.214	85.46	2	30.13	304	30
-122	2.368	68.74	2	20.51	349	35
011	1.245	56.71	2	88.99	924	92
012	1.718	37.29	2	7.36	56	6
021	2.191	71.82	2	243.16	2828	283
022	2.490	56.71	2	49.27	526	53
20-1 + -202	1.505	77.22	1	3345.71	28317	2832
200 + -203	1.785	55.30	1	5271.54	33629	3363
21-1 + -212	1.830	79.52	2	2092.46	22654	2265
210 + -213	2.066	60.54	2	2689.77	26560	2656
220 + -223	2.742	68.25	2	131.01	1822	182
-314	2.719	63.60	2	124.83	2035	203
-315	3.084	52.15	2	451.24	4699	470
-214 + 211	2.487	46.34	2	65.00	461	46
-215 + 212	3.000	36.85	2	163.56	2186	219
-224 + 221	3.071	56.01	2	640.55	6666	667
-225 + 222	3.500	46.69	2	383.16	3986	399
-113	2.000	39.55	2	149.35	1865	187
-114	2.564	29.78	2	278.68	3514	351
-123	2.692	55.06	2	54.02	910	91
013	2.299	26.91	2	10.89	91	9
014	2.925	20.84	2	114.00	1348	135
113	2.858	26.46	2	32.79	280	28
31-1	2.576	70.95	2	974.51	13109	1311
310	2.873	57.95	2	109.60	1569	157

S6. Results fluorapatite

Table S6 Table containing a full list of the calculated intensities I_{calc} of fluorapatite including the Laue indices *hkl* and the reference position in q, ψ and ϕ.

The measured intensities I_{meas} were obtained via 3D numerical integration with integration error ΔI_{meas} from 720 GIXD measurements of a fluorapatite single crystal with incidence angle 1° and a 100 µm pinhole. The results lead to a reliability factor of R = 23.5%.

hkl	q / Å	ψ / °	ϕ / °	I_{calc} / a.u.	I_{meas} / a.u.	ΔI_{meas} / a.u.
010	0.775	60.00	90.00	2409.54	1636	164
1-10	0.775	60.00	270.00	2415.88	1110	111
101	1.198	49.69	180.00	2206.08	1235	124
10-1	1.198	49.69	360.00	2194.79	1182	118
011	1.198	71.13	143.70	2200.41	1254	125
01-1	1.198	71.13	36.30	2200.41	1476	148
1-1-1	1.198	71.13	323.70	2194.79	1104	110
1-11	1.198	71.13	216.30	2206.08	1354	136
110	1.342	30.00	90.00	972.35	1538	154
2-10	1.342	30.00	270.00	972.35	1166	117
020	1.550	60.00	90.00	7768.33	5698	285
2-20	1.550	60.00	270.00	7748.33	2829	141
11-1	1.624	44.28	36.30	6632.66	2725	273
111	1.624	44.28	143.70	6632.67	3221	322
2-1-1	1.624	44.28	323.70	6632.74	2456	246
2-11	1.624	44.28	216.30	6632.74	3584	359
20-1	1.799	30.52	360.00	6246.81	2122	212
201	1.799	30.52	180.00	6212.42	1788	179
02-1	1.799	64.49	55.76	6229.53	3493	349
021	1.799	64.49	124.24	6229.53	2515	252
2-2-1	1.799	64.49	304.24	6246.81	2279	228
2-21	1.799	64.49	235.76	6212.42	3561	356
10-2	1.985	67.02	360.00	14672.22	7574	758
102	1.985	67.02	180.00	14672.44	6417	642
1-1-2	1.985	78.74	339.83	14672.22	6329	633
1-12	1.985	78.74	200.17	14672.44	7775	778
01-2	1.985	78.74	20.17	14659.54	7763	777
012	1.985	78.74	159.83	14659.54	6626	663
120	2.050	40.89	90.00	5910.69	5133	257
3-20	2.050	40.89	270.00	33110.23	10984	549
-130	2.050	79.11	90.00	33072.21	14654	1466
2-30	2.050	79.11	270.00	5934.82	2939	295
12-1	2.244	46.33	55.76	55952.31	13167	1317
121	2.244	46.33	124.24	56000.21	11861	1186
3-2-1	2.244	46.33	304.24	95586.63	17571	1757
3-21	2.244	46.33	235.76	95399.00	21636	2164
20-2	2.396	49.69	360.00	43207.05	12649	1265
202	2.396	49.69	180.00	43207.76	16330	1633
2-2-2	2.396	71.13	323.70	43207.05	11922	596

2-22	2.396	71.13	216.30	43207.76	21158	1058
02-2	2.396	71.13	36.30	43248.43	15082	754
022	2.396	71.13	143.70	43248.43	16647	832
30-1	2.498	21.45	360.00	12747.11	4042	404
301	2.498	21.45	180.00	12747.14	5619	562
220	2.684	30.00	90.00	1011.04	3177	159
4-20	2.684	30.00	270.00	1011.04	2446	122
21-2	2.746	45.14	20.17	11862.21	9003	901
212	2.746	45.14	159.83	11862.56	9263	927
3-1-2	2.746	45.14	339.83	4688.53	6221	622
3-12	2.746	45.14	200.17	4688.20	7724	773
12-2	2.746	55.64	36.30	4701.14	6643	332
122	2.746	55.64	143.70	4701.03	3971	199
3-2-2	2.746	55.64	323.70	11852.00	10810	541
3-22	2.746	55.64	216.30	11852.53	13845	692
130	2.794	46.10	90.00	51293.16	32233	3223
4-30	2.794	46.10	270.00	14938.90	15384	1539
22-1	2.835	34.93	55.76	14254.01	8862	886
221	2.835	34.93	124.24	14253.98	11329	1133
4-2-1	2.835	34.93	304.24	14253.80	6539	654
4-21	2.835	34.93	235.76	14253.80	11353	1136
31-1	2.939	22.68	36.30	8706.23	4527	453
311	2.939	22.68	143.70	8756.56	8224	822
4-1-1	2.939	22.68	323.70	7949.09	7391	739
4-11	2.939	22.68	216.30	8013.23	9076	908
13-1	2.939	48.77	65.59	7973.22	13247	662
131	2.939	48.77	114.41	7989.25	11136	557
4-3-1	2.939	48.77	294.41	8697.90	11757	588
4-31	2.939	48.77	245.59	8764.99	14971	749
30-2	2.957	38.17	360.00	6543.42	6805	340
302	2.957	38.17	180.00	6544.17	6544	327
